# Peripheral facial palsy: contemporary advances and unresolved debates in management from pathogenic mechanisms to tailored reconstruction

**DOI:** 10.3389/fneur.2026.1907653

**Published:** 2026-08-04

**Authors:** Sitao Liang, Yonghua Zhu

**Affiliations:** Department of Neurosurgery, Zhongshan People's Hospital, Zhongshan, Guangdong, China

**Keywords:** Bell’s palsy, botulinum toxin, facial nerve decompression, functional electrical stimulation, multimodal rehabilitation, nerve transfer, neuromodulation, peripheral facial palsy

## Abstract

**Background:**

Peripheral facial palsy (PFP) exhibits highly variable etiology and clinical course. While some patients recover spontaneously within weeks, others develop persistent sequelae including synkinesis and crocodile tears that substantially impair quality of life. Management strategies differ considerably across clinical centers worldwide, reflecting ongoing uncertainty regarding optimal therapeutic approaches.

**Methods:**

This narrative review examines PFP pathophysiology, pharmacotherapy, surgical interventions, PRF neuromodulation, and rehabilitation technologies (125). A literature search of PubMed, Embase, and Cochrane Library (inception to June 2026) was conducted using keywords including “Bell’s palsy,” “peripheral facial palsy,” “facial nerve decompression,” “pulsed radiofrequency,” and “rehabilitation.” RCTs and observational studies were evaluated, with emphasis on systematic reviews and meta-analyses. All pooled statistics are reproduced from cited meta-analyses rather than generated by this review.

**Results:**

Bell’s palsy pathogenesis involves a triple-hit cascade comprising viral reactivation, immune-mediated inflammation, and ischemic edema within the rigid Fallopian canal. Corticosteroids remain the first-line pharmacological intervention; antiviral agents demonstrate incremental benefit only in severe presentations (number needed to treat [NNT] = 15; as reported in a meta-analysis by de Almeida et al.). Surgical decompression for idiopathic facial palsy lacks Level I randomized controlled trial (RCT) evidence. PRF neuromodulation, a minimally invasive interventional modality, occupies an intermediate therapeutic position between pharmacotherapy and open surgery, demonstrating anti-inflammatory and neuroprotective effects with 70–90% symptom relief in hemifacial spasm at 6–24 months. Functional electrical stimulation (FES), extended reality (XR), and artificial intelligence (AI)-enhanced rehabilitation technologies show theoretical promise but await rigorous clinical validation.

**Conclusion:**

PFP management is evolving toward precision medicine, incorporating biomarker-driven patient stratification, AI-assisted outcome prediction, and multinational collaborative trials to establish evidence-based therapeutic standards. Four critical evidence gaps are identified: corticosteroid-antiviral stratification, decompression RCTs, PRF standardization, and rehabilitation validation.

## 1 Introduction

Peripheral facial palsy (1) (PFP) affects 15 to 30 individuals per 100,000 annually (2). Approximately 70% of cases are classified as idiopathic (Bell’s palsy), while the remainder result from identifiable causes including trauma, infection, neoplasia, or iatrogenic injury (3, 4). Although spontaneous recovery occurs in the majority of patients, nearly 30% develop debilitating sequelae including synkinesis, crocodile tears, and persistent facial weakness that profoundly diminish health-related quality of life (5). The clinical heterogeneity of PFP raises two fundamental questions: which patients are at risk for severe or prolonged courses, and what reconstructive strategies are most appropriate once permanent damage has occurred?

Current treatment paradigms for PFP exhibit substantial global variability. Corticosteroids are universally accepted as first-line therapy for acute Bell’s palsy; however, the routine addition of antiviral agents, optimal dosing regimens, and surgical thresholds for facial nerve decompression remain subjects of considerable controversy (6, 7). Chronic reconstruction through selective nerve transfers and free functional muscle grafts has advanced considerably in recent decades, yet standardization remains limited due to operator-dependent variability and the absence of comparative effectiveness trials (8, 9). Rehabilitation approaches are also evolving rapidly, with functional electrical stimulation (FES), extended reality (XR), and artificial intelligence (AI)-based biofeedback systems theoretically capable of enhancing neuroplasticity and improving patient adherence to exercise protocols (10–12). However, definitive evidence from adequately powered randomized controlled trials (RCTs) supporting these technologies over standard physiotherapy remains scarce.

Pulsed radiofrequency (PRF) neuromodulation has emerged as a minimally invasive interventional option for patients who fail conservative management but are unsuitable candidates or unwilling to undergo open surgical procedures (13, 14). Originally developed for chronic pain applications, PRF has demonstrated anti-inflammatory and neuroprotective effects in preclinical models and limited clinical series, with emerging evidence suggesting potential benefits for hemifacial spasm and post-paralytic complications (15, 16). Its hypothesized mechanism as a bridge between pharmacotherapy and surgical reconstruction warrants further prospective validation.

This review systematically examines the current evidence on PFP management, encompassing molecular pathophysiology, acute pharmacotherapy, surgical interventions, PRF neuromodulation, emerging rehabilitation technologies, and global practice variations. Areas of established consensus are highlighted, and ongoing controversies are presented with competing perspectives.

## 2 Methods

This is a narrative review examining peripheral facial palsy pathophysiology, pharmacotherapy, surgical interventions, PRF neuromodulation, and rehabilitation technologies. As a narrative review, this work does not adhere to the Preferred Reporting Items for Systematic Reviews and Meta-Analyses (PRISMA) guidelines, which are specific to systematic reviews with predefined protocols and exhaustive search strategies (17). Instead, the methodology follows established frameworks for narrative reviews, emphasizing thematic synthesis, expert interpretation, and balanced presentation of the evidence (18, 19). The SANRA (Scale for the Quality Assessment of Narrative Review Articles) criteria were used as a guiding framework to ensure methodological rigor (20). A comprehensive literature search was conducted in PubMed, Embase, and Cochrane Library databases from inception to June 2026. The search strategy employed both Medical Subject Headings (MeSH) terms and free-text keywords, including: “Bell’s palsy,” “peripheral facial palsy,” “facial nerve decompression,” “pulsed radiofrequency,” “neuromodulation,” “nerve transfer,” “functional electrical stimulation,” “botulinum toxin,” “synkinesis,” and “rehabilitation.” Boolean operators (AND, OR) were used to combine search terms. No language restrictions were applied; however, English-language articles were prioritized, with selected non-English studies included when methodologically robust.

Both randomized controlled trials (RCTs) and observational studies were evaluated, with emphasis on systematic reviews, meta-analyses, and clinical practice guidelines. Studies were excluded if they: (i) lacked peer review; (ii) were published solely as conference abstracts without full-text availability; (iii) involved fewer than 10 participants without complementary mechanistic or methodological justification; or (iv) were limited to animal models without corresponding clinical data. All pooled statistics (relative risks, confidence intervals, numbers needed to treat) reported herein are reproduced from cited meta-analyses and systematic reviews rather than generated by this review.

This manuscript was prepared in accordance with the Recommendations for the Conduct, Reporting, Editing, and Publication of Scholarly Work in Medical Journals issued by the International Committee of Medical Journal Editors (ICMJE). A formal protocol was not registered, as this is a narrative review rather than a systematic review. The authors independently screened titles and abstracts, with disagreements resolved by consensus. Several references from 2026 lack complete volume, issue, or page numbers; these articles were published online ahead of print with assigned DOIs at the time of manuscript preparation.

## 3 Pathophysiological mechanisms as therapeutic targets

### 3.1 The triple-hit cascade

Bell’s palsy pathogenesis does not result from a single pathogenic event; rather, three converging biological processes drive disease progression (21, 22). Viral reactivation, predominantly herpes simplex virus type 1 (HSV-1) and varicella-zoster virus (VZV), occurs from latency in the geniculate ganglion, with subsequent retrograde migration causing endoneurial swelling and direct viral cytopathic damage (22, 23). While direct viral injury is real, it is considered secondary to the dominant immune-mediated inflammatory response. CD4 + and CD8 + T-cell infiltration releases tumor necrosis factor-alpha (TNF-alpha), interleukin-1 beta (IL-1beta), and other pro-inflammatory mediators, driving vascular permeability and elevating intraneural pressure (24). Trapped within the rigid Fallopian canal, this inflammatory edema compresses the microvasculature, resulting in venous obstruction and reduced arterial perfusion, thereby establishing a vicious edema-ischemia-edema cycle (25). Laser speckle imaging has confirmed that facial nerve perfusion decreases significantly on the affected side, with symmetry indices correlating positively with the severity of clinical deficit (26). Subsequent reperfusion injury generates reactive oxygen species (ROS), lipid peroxidation, and apoptotic cell death (27). This pathophysiological complexity explains why monotherapy approaches, whether antiviral or anti-inflammatory alone, are insufficient to interrupt all three pathogenic links simultaneously (28).

### 3.2 Anatomical vulnerability and individual susceptibility

The meatal segment of the facial nerve, measuring approximately 0.68 mm in diameter, represents the narrowest and most anatomically vulnerable point (22). High-resolution computed tomography (29) (HRCT) reveals substantial interindividual variability in canal diameter, length, and angulation, which explains why equivalent degrees of edema produce divergent clinical outcomes (30). The concept of anatomical susceptibility is exemplified by Jansen metaphyseal chondrodysplasia, in which bilateral facial canal stenosis causes progressive facial paralysis, serving as a clinical model of structural vulnerability (31). Bony narrowing of the internal auditory meatus has also been reported as a rare cause of hemifacial spasm (32). In patients with recurrent PFP, HRCT may reveal exostoses or other bony anomalies that can guide targeted decompressive surgery (33). The observation that some patients experience mild disease while others progress to complete paralysis can be attributed, at least in part, to these volume-pressure dynamics (34). Preoperative imaging biomarkers based on facial canal morphology may one day enable treatment intensity stratification (Table 1).

**Table 1:** Standard PRF parameters for facial nerve applications.

Parameter	Standard setting	Notes
Frequency	2–5 Hz	Lower for neurotrophic induction; higher for hyperexcitability suppression
Pulse width	10–20 ms	Individualized based on electrophysiological thresholds
Temperature	≤42 °C	Non-ablative; structure-preserving
Needle	22-gauge RF cannula	CT or ultrasound-guided placement
Target access	Stylomastoid foramen or trans-parotid branch	Main trunk for hemifacial spasm; terminal branches for focal sequelae
Monitoring	LSR + motor evoked potentials	LSR attenuation correlates with favorable outcome
Outcomes (HFS)	70–90% symptom relief at 6–24 months	Recurrence may require repeat treatment
Safety profile	Favorable	Transient weakness, hematoma; permanent injury rare with image guidance

### 3.3 Molecular regulation of regeneration and aberrant repair

Following axonal injury, distal segments undergo Wallerian degeneration while Schwann cells (35) dedifferentiate and form Bungner bands that guide regenerating axons (36, 37). This process is coordinated by multiple signaling pathways: Hedgehog signaling drives fibroblast migration and vascular endothelial growth factor-A (VEGF-A) upregulation, while differential SMAD1-8 expression between compression and transection injuries suggests pathway-specific therapeutic targets (35, 38). Autophagy, mediated by LC3 and Beclin-1, clears damaged organelles and meets increased energetic demands during regeneration (39). However, regenerative processes frequently result in aberrant reinnervation. Synkinesis occurs when regenerating axons miss their intended targets and innervate adjacent facial muscle groups instead, resulting in involuntary co-contraction (40, 41). Crocodile tears represent parasympathetic fiber misrouting, in which lacrimal gland innervation is supplied by fibers originally destined for salivary glands (40).

These phenomena reflect deficient axonal guidance mechanisms. Exogenous SLIT2 administration and Janus kinase/signal transducer and activator of transcription (JAK/STAT) pathway modulation have shown preclinical promise for improving axonal targeting (10, 42–44). Growth-associated protein 43 (GAP-43) expression and myelin basic protein (MBP) levels serve as biochemical markers of regeneration progress (45, 46). However, both axonal guidance and biochemical monitoring approaches remain in the investigational stage, and clinical translation has not yet been achieved (Table 2).

**Table 2:** Summary of evidence for PFP management modalities.

Modality	Evidence level	Key findings	Limitations
Corticosteroids	High (AAN guidelines)	40–60 mg/day prednisone × 10 days; best within 72 h	Optimal regimen debated; IV vs. PO unresolved
Antivirals	Moderate	Marginal benefit in severe cases (NNT = 15)	Minimal value in unselected populations
Decompression	Low (no Level I RCTs)	ENoG >90% within 14 days as threshold	Hearing loss risk 1–3%; evidence gap urgent
Nerve transfer	Moderate-High	Masseteric-facial: faster recovery, fewer synkinesis	Smile requires mastication; cortical plasticity variable
FFMT (gracilis)	Moderate	Gold standard for >1 year with atrophy	Long recovery; two-stage for CFNG
PRF neuromodulation	Emerging	70–90% HFS relief; non-ablative; safe	Standardized protocols lacking; long-term RCTs needed
FES rehabilitation	Low-Moderate	Counters atrophy; improves smile angle	Synkinesis risk; parameter standardization lags
XR/AI rehab	Preliminary	Enhanced adherence; telerehab feasible	No well-powered long-term RCTs

### 3.4 Translating mechanistic insights into therapeutic targets

Precision medicine in PFP requires simultaneous targeting of multiple pathophysiological nodes. Corticosteroids effectively interrupt the inflammatory-edema loop but lack antiviral activity, whereas acyclovir inhibits HSV-1 replication yet provides benefit only in select patient subgroups (6). JAK/STAT inhibitors offer dual-target anti-inflammatory and immunomodulatory appeal, but robust clinical data are currently lacking (44). Low-level laser therapy has demonstrated oxidative stress modulation with elevated superoxide dismutase (SOD) and reduced malondialdehyde in early-phase trials (11). Magnesium alloy-based bone synthesis scaffolds with neural guidance channels represent an emerging tissue engineering approach, though clinical application remains distant (12). A common bottleneck across all three therapeutic modalities is the gap between animal model findings and human clinical translation, compounded by the absence of validated predictive biomarkers and high bench-to-bedside attrition rates (47).

## 4 Acute-phase pharmacotherapy: evidence and controversy

### 4.1 Corticosteroids

Corticosteroids constitute the evidence-based foundation for acute Bell’s palsy management. Systematic reviews and clinical practice guidelines from the American Academy of Neurology (AAN) and the American Academy of Otolaryngology-Head and Neck Surgery (AAO-HNS) have established strong recommendations for corticosteroid use based on landmark RCT evidence (6, 48, 49). Three foundational randomized controlled trials support this recommendation: the Bells trial (Sullivan et al., N = 551) demonstrated that prednisolone significantly increased recovery rates at 3 and 9 months versus placebo (50); the Scandinavian trial (Engström et al., *N* = 839) confirmed improved facial function recovery with prednisolone alone or combined with valaciclovir (51); and the Japanese trial (Hato et al., *N* = 221) supported combination therapy with valacyclovir and prednisolone for severe presentations (52). Prednisone-equivalent dosing of 40 to 60 mg daily for 10 days has demonstrated superiority over placebo, and corticosteroid monotherapy outperforms antiviral monotherapy (53). The optimal regimen remains debated: intravenous dexamethasone at 10 mg daily for 5 days followed by taper, when combined with facial rehabilitation, has shown favorable outcomes (54). The comparative efficacy of high-dose pulse versus standard-dose corticosteroid regimens remains unresolved (48). One randomized controlled trial reported that combination therapy (corticosteroids plus antivirals) improved mean House-Brackmann scores from 3.1 to 1.1 at six months; however, the specific contribution of corticosteroids cannot be isolated from this finding (55). The timing of treatment initiation is critical: administration within 72 h of symptom onset maximizes therapeutic benefit, with efficacy declining substantially thereafter (6, 56). Early treatment also reduces the incidence of sequelae including crocodile tears and synkinesis (57).

### 4.2 Antiviral combinations

The evidence supporting antiviral adjunctive therapy remains equivocal. Early small-scale studies suggested benefit from combination therapy; however, subsequent Cochrane reviews have demonstrated minimal incremental value over corticosteroids alone in unselected populations (53, 58). Stratified analysis reveals a different pattern: in patients with complete paralysis or severe presentations, combination therapy confers modest benefit, with a number needed to treat (NNT) of 15 for incomplete recovery and 12 for synkinesis prevention [as reported in the meta-analysis by de Almeida et al. (57)]. These benefits are fragile and restricted to specific subgroups. For Ramsay Hunt syndrome, which is varicella-zoster virus-mediated, intravenous corticosteroids plus antiviral therapy is the established standard of care, with early treatment being critical (59–61). In Bell’s palsy without virological confirmation, universal combination therapy lacks a robust evidence base (23).

Clinical guidelines reflect this uncertainty: the AAN and AAO-HNS advocate corticosteroid monotherapy (48, 49), while Korean guidelines conditionally endorse combination therapy for severe or virologically suspected cases (6). The Japanese Otological Society recommends antiviral addition for zoster-suspected cases (62). These divergent recommendations reflect differences in risk tolerance and healthcare system structures rather than fundamental disagreement regarding the underlying evidence. The persistent debate underscores the need for better etiological stratification tools to identify patients most likely to benefit from antiviral adjuncts.

### 4.3 Neurotrophic and adjunctive agents

Methylcobalamin (63) monotherapy has demonstrated limited clinical effectiveness in PFP (64). However, when combined with silicon-derived antioxidants, enhanced remyelination, improved functional restoration, and reduced oxidative damage have been observed in preclinical models (65). Prostaglandin E1 has theoretical neural perfusion augmentation properties, but clinical data are absent, limiting its use to empirical adjunctive status (66). Nerve growth factor (NGF) and brain-derived neurotrophic factor (BDNF) are regenerative in animal models; however, clinical utility is impeded by rapid systemic clearance and the inability to achieve therapeutic concentrations at target sites (67–69). Chitosan-mediated local delivery systems and tacrolimus (FK506) are under investigation, but concerns regarding systemic toxicity limit translation to clinical practice (70).

Intravenous immunoglobulin (IVIG) has been proposed as an alternative for steroid-resistant or contraindicated cases, with small case series reporting variable outcomes (71, 72). Hyperbaric oxygen therapy, though mechanistically rational for ischemic nerve injury, lacks robust clinical trial evidence (73, 74). The common theme across all adjunctive pharmacological approaches is the substantial gap between theoretical benefit and clinically proven efficacy.

### 4.4 Guidelines and east Asian practices

East Asian clinical practice integrates traditional and modern therapeutic approaches. Chinese acupuncture modalities, including floating needle and electroacupuncture techniques, are widely employed. A meta-analysis confirmed that electroacupuncture (75) achieves superior recovery rates compared to manual needling [relative risk [RR] 1.16, 95% confidence interval [CI] 1.11 to 1.22; as reported by Li et al. (42)] and improved functional scores (standardized mean difference [SMD] 2.26, 95% CI 0.15 to 4.37) (76). Functional magnetic resonance imaging (fMRI) studies have linked acupuncture to activation of the cerebellar amygdala and superior frontal gyrus (76). Jin-needle (77) acupuncture has demonstrated superior effectiveness, cure rates, and Facial Disability Index (FDI) scores compared to conventional techniques (73). Fire-needle therapy (78) improves both Sunnybrook and FDI assessments (73).

A network meta-analysis concluded that the combination of acupuncture, massage, and cupping therapy (79) represents the optimal multimodal traditional Chinese medicine approach (80). In Korea and Japan, proprioceptive neuromuscular facilitation (PNF) combined with electrical stimulation is commonly prioritized (54). Integration of these approaches into international clinical practice guidelines remains limited by methodological heterogeneity in published studies, small sample sizes, and the absence of long-term randomized controlled trials (81, 82). Western clinical guidelines emphasize electrophysiological assessment using electroneuronography (ENoG) and electromyography (EMG); however, these modalities are frequently unavailable in resource-limited settings, contributing to a widening evidence-practice gap (6, 83).

## 5 Surgical interventions for peripheral facial nerve injury

### 5.1 Facial nerve decompression

Indications for facial nerve decompression are well established in the context of trauma, cholesteatoma (84), schwannoma, and iatrogenic injury (85–87). However, the role of decompression in idiopathic Bell’s palsy remains a subject of considerable debate. Electroneuronography (ENoG) demonstrating greater than 90% denervation within 14 days of complete paralysis onset remains the most widely accepted surgical threshold (88, 89). The rationale for decompression is that the facial nerve swells within the non-expandable Fallopian canal, and surgical decompression interrupts the edema-ischemia cycle described in Section 2 (90).

Technical approaches vary in invasiveness and anatomical exposure. The transmastoid (91) approach provides familiar otologic access and is adequate for decompression of the vertical and tympanic segments (90). The middle cranial fossa approach exposes the meatal segment and internal auditory canal, which is critical given that this region represents the most anatomically vulnerable segment (92). Endoscopic transcanal approaches offer minimally invasive access with improved visualization of the geniculate ganglion and labyrinthine segment, though they are associated with longer operative times and steeper learning curves (93, 94). Complications include sensorineural hearing loss (1 to 3%), dizziness, and tympanic membrane perforation, which are non-trivial risks (95). The absence of Level I randomized controlled trials supporting facial nerve decompression for Bell’s palsy represents the field’s most urgent evidence gap (96, 97).

### 5.2 Surgical decision-making in traumatic PFP

In traumatic facial palsy, mechanism of injury and timing of presentation are the principal determinants of surgical (98) strategy (99). Temporal bone (100) fractures involving the otic capsule carry a worse prognosis than longitudinal fractures (101, 102). Total versus partial paralysis is differentiated using intraoperative evoked electromyography (EMG), which distinguishes neurapraxia (conduction block) from axonotmesis (axon disruption) or neurotmesis (complete nerve transection), thereby guiding the choice between decompression and grafting (103). Transection in the parotid gland from penetrating trauma requires immediate repair, as outcomes decline sharply when intervention is delayed beyond 72 h (104). Iatrogenic injury during parotidectomy or middle ear surgery necessitates intraoperative identification and direct repair for optimal outcomes (105). Penetrating trauma to the parotid-masseteric region carries a particularly high risk of facial nerve injury (106). The key principle is that electrophysiological assessment must be performed within strict timeframes; delay converts recoverable injury into permanent deficit.

### 5.3 Nerve repair and grafting

For minimal gap transections, tension-free end-to-end neurorrhaphy remains the gold standard (107). Emerging techniques include epineurial (108) and perineurial microsutures with polyethylene glycol (PEG) (109) adjuncts, which have restored acute electrophysiological conduction in porcine models (110). Larger gaps require interposition grafting. The sural nerve is the most commonly used donor, providing superficial access, 30 to 40 cm segments, and appropriate diameter match; lateral dorsal foot numbness is the primary donor site morbidity, which is usually well tolerated (111). The great auricular nerve is suitable for grafting in the parotid region, while the medial antebrachial cutaneous (MABC) nerve can be used even in irradiated surgical fields (112).

Allografts and bioengineered conduits remain investigational. Decellularized allografts (113) have demonstrated 85% functional recovery in facial nerve defects less than 50 mm; however, longer gap reconstructions have been unsuccessful (114). Polyglycolic acid (PGA) tubes have shown partial restoration and neuronal death reduction (115). Bioengineering approaches incorporating growth factor-coated scaffolds and 3D-printed nerve guides are in early preclinical development (116). Robust clinical evidence supporting these bioengineered alternatives remains limited, and sural nerve autografting continues to be the clinical standard for gap reconstruction.

### 5.4 Nerve transfers

Irreversible proximal facial nerve lesions require nerve transfer procedures to restore facial motor function. The hypoglossal-to-facial nerve transfer (cranial nerve XII-to-VII) provides robust motor drive; however, traditional end-to-end anastomosis is associated with significant morbidity, including tongue atrophy in 73% of patients, as well as dysphagia and dysarthria (117). End-to-side and split-nerve modifications that preserve hypoglossal nerve continuity have been developed to address this limitation, achieving dramatically reduced tongue atrophy with equivalent facial recovery (118). Masseteric-to-facial nerve transfer has emerged as an alternative with several advantages: faster regeneration time (4.6 versus 6.3 months), stronger commissure displacement, and fewer synkinetic movements (119). The primary trade-off is that smile initiation requires masticatory effort; however, cortical plasticity may enable spontaneous smiling in a subset of patients (120).

Accessory nerve transfer has been largely abandoned because of associated shoulder dysfunction and is now reserved for last-resort scenarios (121, 122). Pediatric patients demonstrate particular benefit from nerve transfer due to enhanced neuroplasticity (123). Cross-face nerve grafting (CFNG) with simultaneous masseteric nerve transfer represents the current frontier, potentially combining spontaneous emotional expression with reliable motor drive (124). Intraoperative monitoring of compound muscle action potentials (CMAP) during nerve transfer surgery helps optimize tension and alignment, improving functional outcomes (125).

### 5.5 Muscle reconstruction

In patients with denervation exceeding one year and significant muscle atrophy, nerve repair alone is insufficient for functional restoration. Free functional muscle transfer (FFMT) (126), most commonly utilizing the gracilis muscle (127), provides reliable neurovascular supply and adaptable tissue bulk (128). Reinnervation strategy influences functional outcomes: cross-face nerve (129) grafting (CFNG) enables spontaneous, emotionally synchronized smiling but requires a two-stage approach with prolonged recovery; masseteric nerve transfer allows single-stage reconstruction with more rapid return of movement, though voluntary jaw movement is required to trigger the smile (130). Dual innervation combining CFNG and masseteric nerve input integrates the advantages of both approaches (131). Segmental gracilis harvest can simultaneously correct lagophthalmos (132).

For larger soft tissue defects, the latissimus dorsi muscle provides greater volume; the pectoralis minor better mimics native facial architecture but has a shorter vascular pedicle (133, 134). In elderly or medically complex patients who cannot tolerate free tissue transfer, static suspension with tensor fascia lata has demonstrated satisfactory commissure positioning (135). Temporalis muscle transposition avoids the need for microvascular anastomosis but, like masseteric transfer, requires masticatory effort for smile activation (136). Contemporary facial reanimation has shifted from isolated procedures toward multimodal reconstruction, with combined approaches incorporating muscle transfer, FFMT, and static slings addressing multidimensional deficits in severe cases (137, 138).

While surgical reconstruction provides the anatomical framework, not all patients are suitable candidates for or willing to undergo open operative procedures. Furthermore, post-paralytic synkinesis affects 9–55% of patients recovering from facial nerve injury and requires targeted management through botulinum toxin injection and neuromuscular retraining (Section 4.6). For patients with neuronal hyperexcitability rather than structural nerve discontinuity, minimally invasive neuromodulation strategies such as pulsed radiofrequency offer an alternative therapeutic avenue, as detailed in the following section.

### 5.6 Management of post-paralytic synkinesis

Post-paralytic synkinesis, occurring in 9–55% of patients recovering from facial nerve injury, represents (139) aberrant reinnervation where regenerating axons innervate unintended muscle groups, resulting in involuntary co-contraction during volitional movement (140). The orbicularis oculi, platysma, mentalis, and perioral muscles are most commonly affected. Synkinesis substantially impairs quality of life and may worsen over time without intervention.

#### 5.6.1 Botulinum toxin injection

Botulinum toxin type A (BTX-A) injection is the cornerstone of synkinesis management. By blocking acetylcholine release at the neuromuscular junction, BTX-A selectively weakens hyperactive synkinetic muscles while preserving voluntary function on the affected side (141). Injection to the contralateral healthy side can additionally improve facial symmetry at rest and during animation. Commonly targeted muscles include the platysma (10–30 units), orbicularis oculi (2.5–7.5 units), mentalis (2.5–10 units), and depressor anguli oris (2.5–10 units) on the affected side, with frontalis (5–10 units) and depressor labii inferioris (2.5–5 units) frequently injected on the unaffected side to achieve symmetry (141). Treatment intervals typically range from 4 to 8 months. A systematic review and meta-analysis of BTX-A for facial palsy synkinesis demonstrated significant improvements in synkinesis scores and facial symmetry with favorable safety profiles (142).

#### 5.6.2 Neuromuscular retraining

Neuromuscular retraining (NMR) programs emphasize selective motor control through slow, symmetrical facial movements with mirror biofeedback, teaching patients to isolate intended muscles while avoiding compensatory recruitment of synkinetic groups (143). Combination therapy integrating BTX-A injection followed by structured NMR has demonstrated superior outcomes compared to either modality alone, with sustained improvement persisting beyond the pharmacological duration of toxin effect (144). Clinical practice guidelines from the International Head and Neck Scientific Group recommend combined BTX-A and NMR as first-line therapy for moderate-to-severe synkinesis (140).

For refractory cases unresponsive to BTX-A and NMR, modified selective neurectomy may be considered. This technique involves targeted transection of hyperactive terminal nerve branches supplying synkinetic muscles, with published reports demonstrating long-term reduction in botulinum toxin dependency (145).

## 6 Minimally invasive neuromodulation: pulsed radiofrequency

### 6.1 Mechanistic foundations

A substantial proportion of patients with post-paralytic facial (146) nerve disorders fall into a therapeutic gap: their symptoms are too severe for conservative management, yet they are either not severe enough or unwilling to undergo open surgical intervention. Pulsed radiofrequency (PRF) delivers pulsed electrical fields at 2 to 5 Hz with 10 to 20 millisecond pulse width, maintaining tissue temperature below 42 degrees Celsius, thereby preserving structural nerve integrity while modulating neuronal excitability (14). Unlike continuous radiofrequency thermocoagulation, which produces thermal nerve injury, PRF has been proposed as a non-ablative intermediate therapy for selected facial nerve disorders (15). However, the evidence supporting PRF in cranial nerve applications remains predominantly derived from small case series rather than randomized controlled trials (16).

Preclinical studies suggest that PRF modulates voltage-gated sodium and calcium channels, dampening abnormal ectopic firing, and may alter synaptic transmission with concurrent changes in neuronal excitability (15, 16, 147). Whether these mechanisms translate into sustained clinical improvement in facial nerve disorders remains to be confirmed in well-controlled clinical trials. In pain and motor control pathways have been documented following PRF exposure, which may explain the persistence of clinical improvement beyond the immediate procedural period (16).

The anti-inflammatory and nerve-protective properties of PRF are of particular relevance to facial nerve disorders. Following injury, pro-inflammatory cytokines including TNF-alpha, IL-1beta, and interleukin-6 (IL-6) exacerbate endoneurial edema and impede regeneration. PRF has been demonstrated to suppress these pro-inflammatory mediators while upregulating brain-derived neurotrophic factor (BDNF) and nerve growth factor (NGF) (148). This dual mechanism, simultaneously reducing inflammation while promoting neurorepair, addresses pathophysiological targets that neither corticosteroids nor antiviral agents adequately cover. Early clinical data indicate not only symptomatic relief but measurable functional score improvements, which correlate with reductions in inflammatory markers and elevations in neurotrophic factors (148). In appropriately selected patients, PRF may modify the disease course rather than merely providing palliative symptom control.

### 6.2 Anatomical targets and techniques

The complex anatomical course of the facial nerve, exiting the stylomastoid foramen (149), arborizing within the parotid gland (150) into the pes anserinus, and subsequently innervating the mimetic musculature, necessitates precise targeting (151). Two principal access routes are the stylomastoid foramen approach (main trunk, for hemifacial spasm) and the trans-parotid branch approach (selective terminal branches, for focal sequelae) (15). CT or ultrasound guidance is employed with a 22-gauge cannula; tip position is confirmed by low-current stimulation eliciting facial contraction. Irrespective of the operator must avoid injury to Stensen’s duct and adjacent vascular structures, as salivary fistula and hematoma are recognized procedural risks (152).

Intraoperative (106) lateral spread response (LSR) monitoring guides PRF delivery; attenuation of LSR correlates with favorable clinical outcomes (153). Direct motor evoked potential recording provides real-time verification of electrode position.

### 6.3 Parameter optimization

Standard PRF parameters of 2 to 5 Hz frequency, 10 to 20 millisecond pulse width, and maximum tissue temperature of 42 degrees Celsius represent starting points rather than optimized therapeutic values (14, 154). For hemifacial spasm, higher voltages suppress ectopic hyperexcitability; for post-paralytic sequelae, lower intensity is preferred (16). Follow-up at 1, 3, and 6 months using House-Brackmann and Sunnybrook scales guides repeat treatment decisions (151).

### 6.4 Indications and comparative effectiveness

PRF neuromodulation occupies an intermediate therapeutic position between failed pharmacological management and invasive surgical intervention. Current evidence supports three principal indications: (i) primary hemifacial spasm in (155) patients who decline or are unsuitable candidates for microvascular decompression; (ii) post-paralytic sequelae including synkinesis, contracture, and neuropathic pain; and (iii) refractory neuropathic pain following Ramsay Hunt syndrome or trauma (156). Clinical outcomes demonstrate 70 to 90% symptom relief at 6 to 24 months for hemifacial spasm, though recurrence requiring repeat treatment is not uncommon (156).

Compared to botulinum toxin (157), PRF offers more sustained effect (months to >1 year versus 3–4 months) (156). Compared to microvascular decompression, PRF avoids craniotomy-related risks with shorter recovery (156). Unlike selective neurectomy, PRF preserves baseline neural function (15). PRF has also been used for refractory Ramsay Hunt neuralgia (158). Contraindications include active infection, uncorrected coagulopathy, and anatomical variants precluding safe cannula trajectory (159, 160).

### 6.5 Future directions

Future development priorities for PRF in facial nerve disorders include: (i) parameter individualization based on patient-specific electrophysiological signatures (15); (ii) multimodal combination approaches pairing PRF with neurotrophic factor delivery or biofeedback-based rehabilitation (15); and (iii) improved needle placement precision through high-resolution ultrasound and three-dimensional navigation (160). Transitioning PRF from an empiric technique to an evidence-based, mechanism-driven modality will require well-powered randomized controlled trials with standardized protocols and long-term outcome assessment.

It is important to recognize that neuromodulation alone cannot address the biomechanical deficits resulting from prolonged denervation; cortical reorganization and functional recovery still require structured rehabilitation. The role of conventional and technology-enhanced rehabilitation in complementing surgical and interventional modalities is addressed in the following section.

## 7 Rehabilitation and emerging technologies

### 7.1 Conventional rehabilitation

Facial neuromuscular retraining (fNMR) remains the cornerstone of rehabilitation, emphasizing restoration of facial symmetry, coordination of volitional movement, and control of synkinetic activity through structured active exercise protocols (10, 12). Mirror therapy, in which the patient observes the unaffected side to stimulate contralateral cortical activation, has demonstrated improvements in facial symmetry and long-term reduction of synkinesis (140). The combination of proprioceptive neuromuscular facilitation (PNF) with mirror therapy has been associated with enhanced acute-phase recovery scores and shorter time to functional improvement (54). Biofeedback-based training enables patients to visualize their facial muscle activity, improving motor learning and coordination (161).

In Asian clinical settings, acupuncture and massage (162) are widely utilized. Jin-needle acupuncture has demonstrated superior effectiveness, cure rates, and Facial Disability Index (FDI) scores compared to conventional techniques (73). Fire-needle therapy improves both Sunnybrook and FDI assessments (42). A network meta-analysis concluded that the combination of acupuncture, massage, and cupping therapy represents the optimal multimodal traditional Chinese medicine approach (80). Integration of these techniques into international clinical practice guidelines remains limited by methodological heterogeneity in published studies, small sample sizes, and the absence of long-term randomized controlled trials (81, 82).

### 7.2 Functional electrical stimulation

Functional electrical stimulation (FES) targeting the zygomaticus major muscle increases muscle cross-sectional area and counters denervation-induced atrophy (163). Home-based FES systems for complete facial paralysis have demonstrated safety and feasibility, with associated improvements in smile angle and subjective functional ratings (6). Treatment efficacy is influenced by etiology, stimulation parameters, and timing of intervention; synkinesis induction or worsening represents the primary limiting risk (143). Implantable FES systems following hypoglossal-facial neurorrhaphy enable more precise modulation, and systematic reviews have reported functional improvement without significant synkinesis worsening, though parameter standardization across centers remains incomplete (11, 12). The “bionic blink” closed-loop system, in which contralateral electromyographic (EMG) activity triggers affected-side stimulation, represents an exemplar of intelligent neurostimulation technology (161). Closed-loop FES systems that adapt stimulation parameters in real time based on patient response show particular promise for optimizing outcomes while minimizing adverse effects.

### 7.3 Extended reality and artificial intelligence

Extended reality (XR) encompassing virtual reality (VR), augmented reality (AR), and mixed reality (MR) creates novel rehabilitation paradigms by providing immersive, controllable environments for repetitive task-oriented exercise (40). VR-based mirror visual feedback systems project unaffected-side animation onto the impaired hemiface, potentially reactivating quiescent cortical pathways. Biofeedback combined with gamification converts EMG signals into visual and auditory cues, enabling patients to control virtual elements through facial expression, thereby transforming repetitive exercise protocols into engaging interactive activities (161). Telerehabilitation platforms incorporating home-based EMG biofeedback have demonstrated feasibility, with patients able to operate systems independently and sustain exercise intensity (10).

The integration of artificial intelligence (AI) with XR platforms enables adaptive feedback, in which algorithms analyze motion capture and EMG data to modulate exercise difficulty and forecast functional outcomes. Deep learning models for automated facial palsy severity grading have achieved accuracy comparable to expert clinicians (11). However, the current evidence base for XR-based facial palsy rehabilitation remains equivocal. Large-scale, long-term randomized controlled trials specifically targeting facial palsy populations are scarce, with existing studies limited by small sample sizes and short follow-up durations. Generalizability and cross-population adaptability require further validation before these technologies can be integrated into standard care pathways.

### 7.4 Regenerative approaches

Mesenchymal stem cells (MSCs) offer potential therapeutic benefits through neurotrophic factor secretion and inflammatory suppression; however, clinical application remains largely preclinical or in early-phase investigation (47). Toll-like receptor (TLR) expression modulation via gene therapy, informed by observed post-injury alterations in TLR2-5 and TLR7 expression, provides a theoretical basis for future intervention (35). Tissue engineering approaches, including graphene-chitosan nerve conduits and stem cell-loaded scaffolds, show preclinical potential but await clinical validation (114–116). The common barrier across all regenerative approaches is the extended timeline from conceptualization to clinical implementation, compounded by regulatory hurdles, treatment costs, and the steep learning curves associated with emerging technologies. At present, these modalities should be considered adjunctive rather than replacement therapies.

The adoption of rehabilitation technologies varies substantially across regions, shaped by resource availability, cultural factors, and local evidence traditions. Western and Asian clinical landscapes differ in their emphasis on technological versus traditional rehabilitation approaches; the role of multidisciplinary collaboration in bridging these differences is examined in the following section.

## 8 Global perspectives and multidisciplinary collaboration

### 8.1 Western versus Asian models

Western clinical practice generally emphasizes aggressive surgical intervention for refractory cases or definitive nerve transection. Electroneuronography demonstrating greater than 90% denervation typically prompts operative intervention within eight days, with outcomes reported to exceed non-operative management in appropriately selected patients (25, 164). Early adoption of functional electrical stimulation and technology-enhanced rehabilitation reflects a commitment to technological innovation. In contrast, Asian practice integrates traditional and modern approaches synergistically. In China, floating needle and electroacupuncture techniques are widely employed, with meta-analytical evidence supporting the superiority of electroacupuncture over manual needling (76). Qianzheng San, a traditional Chinese medicine formulation, has been shown to promote facial nerve regeneration through BDNF-tropomyosin receptor kinase B (TrkB)-CREB pathway activation (83). In Korea and Japan, proprioceptive neuromuscular facilitation combined with electrical stimulation is commonly prioritized (54). These represent complementary rather than hierarchical differences: Western practice emphasizes precise timing and targeted intervention, while Asian practice prioritizes holistic modulation and functional rehabilitation.

### 8.2 Multidisciplinary teams

Effective management of facial nerve disorders requires multidisciplinary teams encompassing neurology, otolaryngology, plastic surgery, rehabilitation medicine, ophthalmology, and psychology. Middle ear cholesteatoma with associated facial palsy, for example, necessitates collaborative otologic-neurosurgical decompression (85). Following vestibular schwannoma resection, tailored nursing interventions including facial exercises, massage, and video-based education have been shown to improve recovery trajectories and quality of life (86). Pediatric (165) facial palsy presents unique challenges requiring coordinated input from pediatricians, pediatric neurologists, and rehabilitation specialists (87). The transition from specialty-siloed care to integrated multidisciplinary models represents a critical step toward comprehensive management from diagnosis through long-term follow-up.

### 8.3 Decision-making framework

Clinical decision-making in PFP requires integration of multiple patient-specific factors including etiology, disease severity, injury type, temporal stage, and individual patient characteristics. For Bell’s palsy, corticosteroids constitute first-line therapy, with antiviral adjuncts considered in severe presentations. Traumatic facial palsy necessitates electroneuronography-guided surgical decisions within strict timeframes (25, 164). Complete nerve discontinuity warrants primary neurorrhaphy or nerve transfer; partial injuries may be managed conservatively or with structured rehabilitation. The acute phase focuses on suppression of inflammation and reduction of edema; the recovery phase emphasizes axonal regeneration and active training; the sequela phase targets management of synkinesis and contracture. In pediatric populations, prognosis is generally favorable, and the neutrophil-to-lymphocyte ratio (NLR) at admission has been identified as a predictor of outcomes (87). The integration of genomics, proteomics, and advanced neuroimaging for biomarker-guided prognosis and treatment optimization represents an emerging frontier in personalized facial nerve care.

## 9 Discussion

**Figure 1:**
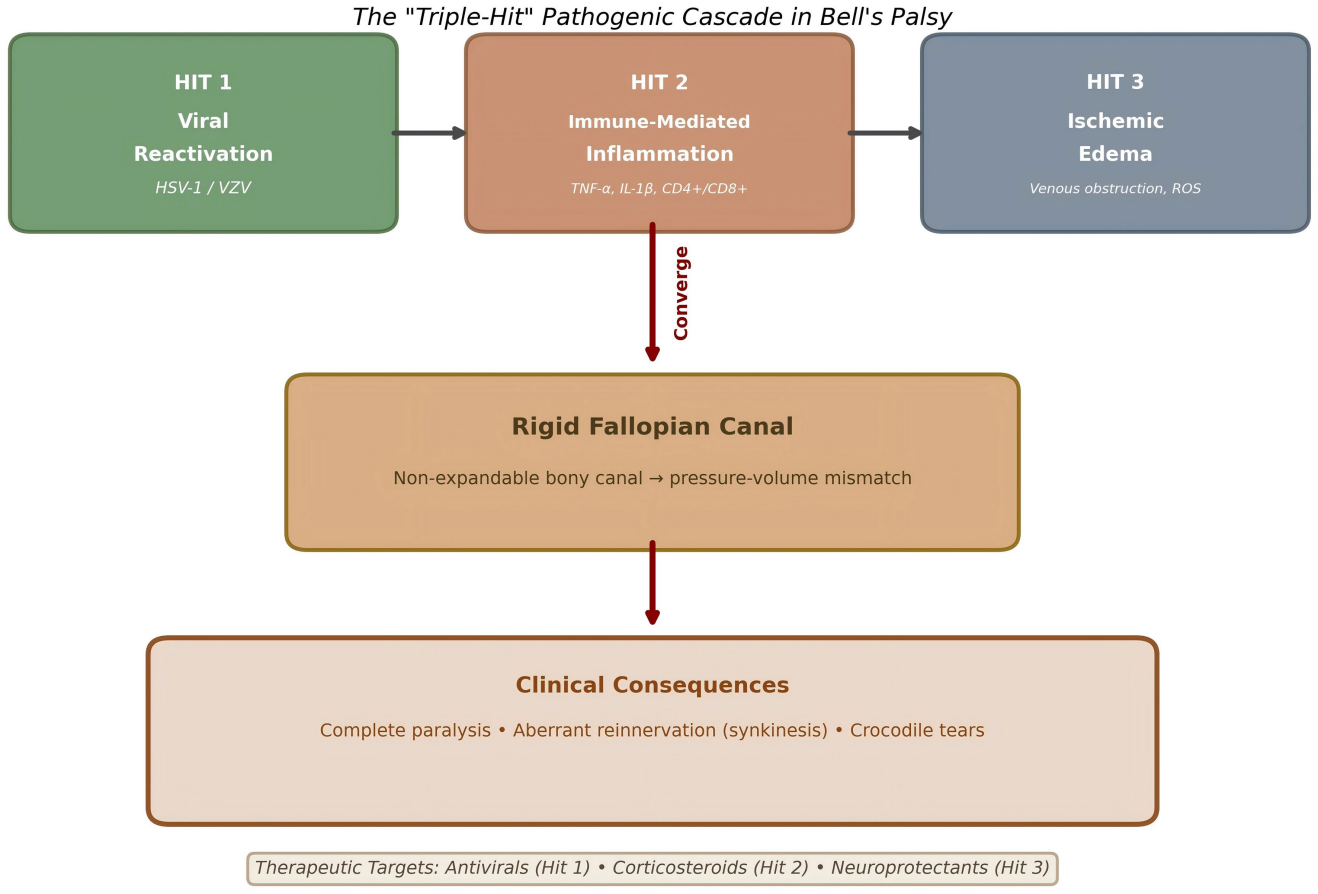
The “triple-hit” pathogenic cascade in Bell’s palsy. Viral reactivation (Hit 1), immune-mediated inflammation (Hit 2), and ischemic edema (Hit 3) converge within the rigid Fallopian canal, producing a self-amplifying pressure-volume mismatch that drives clinical progression. Corresponding therapeutic targets are indicated for each pathogenic node. HSV-1 = herpes simplex virus type 1; VZV = varicella-zoster virus; TNF-*α* = tumor necrosis factor-alpha; IL-1β = interleukin-1 beta; ROS = reactive oxygen species.

This review has identified four principal consensus points that define the current state and future direction of PFP management. Figure 1 schematically illustrates the triple-hit pathogenic cascade that underlies Bell’s palsy, providing a mechanistic framework for understanding current and emerging therapeutic targets.

First, corticosteroids remain the evidence-based pharmacological foundation for acute PFP, with optimal outcomes achieved when treatment is initiated within 72 h of symptom onset (6, 56). The adjunctive use of antiviral agents requires better etiological stratification to identify the specific patient subgroups most likely to benefit, as universal combination therapy is not supported by current evidence in unselected populations (53, 57). The persistent divergence in international guidelines regarding antiviral use underscores the need for validated biomarkers capable of distinguishing viral-mediated from non-viral etiologies at the point of care.

Second, surgical management is evolving toward minimally invasive, function-preserving approaches with enhanced intraoperative electrophysiological monitoring. However, the absence of Level I randomized controlled trial evidence supporting facial nerve decompression for Bell’s palsy represents an urgent and critical evidence gap that must be addressed through adequately powered prospective trials (96, 97). Current operative decisions rely heavily on electrophysiological thresholds, principally ENoG greater than 90% denervation within 14 days (88, 89), but these thresholds have not been prospectively validated against clinical outcomes in controlled studies.

Third, PRF neuromodulation offers a rational intermediate therapeutic option for patients who fail pharmacological management but are unsuitable candidates for invasive surgery, particularly in hemifacial spasm and post-paralytic sequelae. Its non-ablative mechanism, favorable safety profile, and dual anti-inflammatory-neuroprotective properties support expanded clinical evaluation (14–16). However, standardized treatment protocols, long-term comparative effectiveness data, and definitive RCTs comparing PRF to botulinum toxin and microvascular decompression are required before PRF can be fully integrated into clinical practice guidelines. The development of patient-specific electrophysiological signatures to guide parameter individualization represents a promising avenue for improving treatment precision (15).

Fourth, technology-enhanced rehabilitation modalities including FES, XR, and AI-based biofeedback systems demonstrate substantial potential for improving patient adherence and enabling remote delivery of care. However, definitive evidence regarding their impact on long-term functional outcomes remains scarce and will require rigorous multinational validation through adequately powered randomized controlled trials (161, 163, 166, 167). The common theme across all emerging rehabilitation technologies is the gap between theoretical promise and clinically proven efficacy—a pattern that has historically characterized the field of neurorehabilitation.

Two principal limitations constrain the field. The complexity of PFP pathophysiology, with viral, immune, and ischemic layers interacting in ways that outpace single-target therapeutic strategies, means that current interventions frequently fail to prevent sequelae (22, 24, 28). Optimized multi-target combination studies spanning pharmacological, interventional, and rehabilitative domains remain scarce. Second, global practice variations and evidence heterogeneity impede the development of standardized international guidelines. East–West differences in surgical thresholds, rehabilitation timing, traditional medicine integration, and neuromodulation adoption reflect not merely cultural divergence but a fundamental shortage of high-quality multinational randomized controlled trials (76, 81, 82).

**Figure 2:**
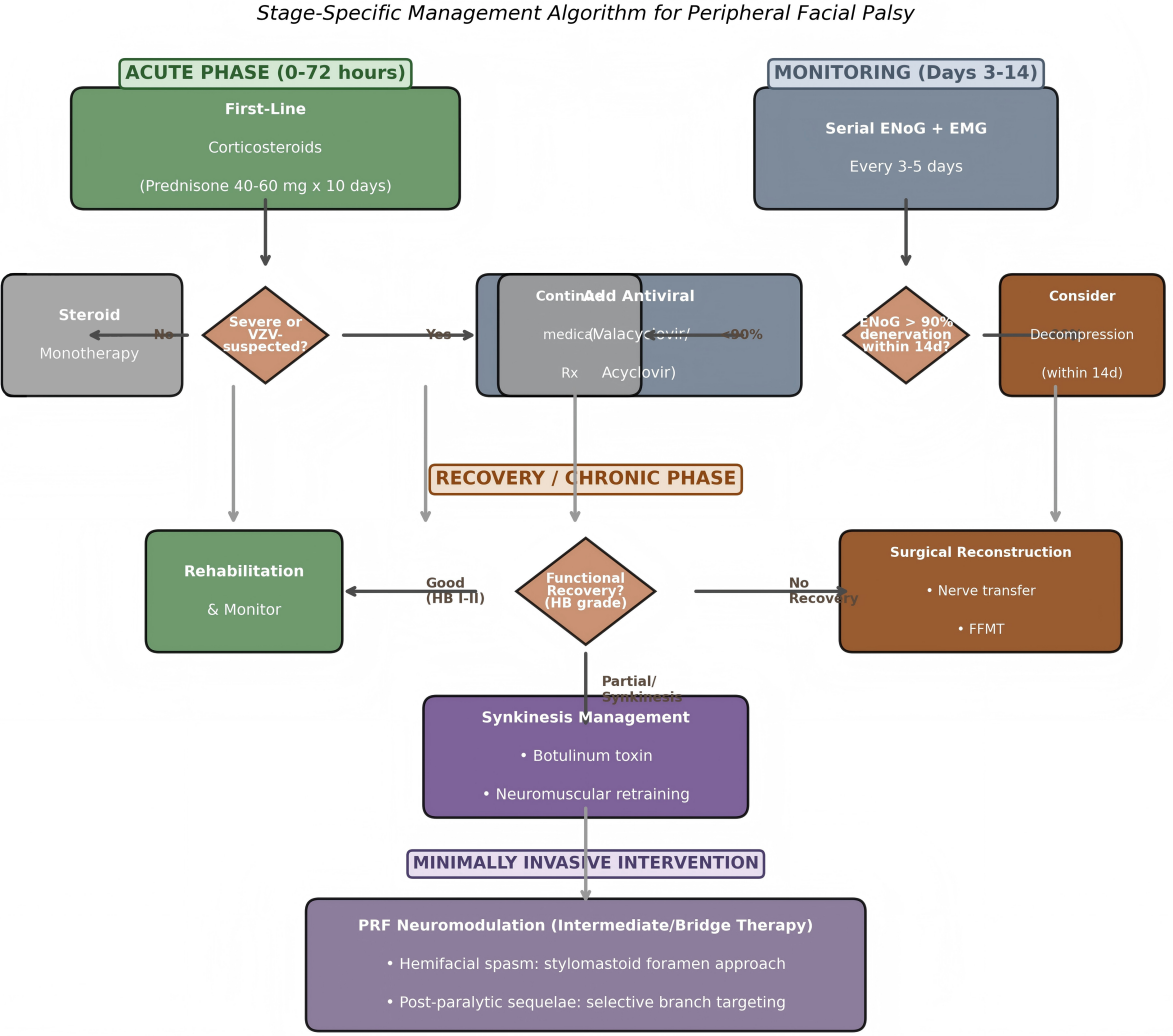
Proposed stage-specific management algorithm for peripheral facial palsy. ENoG = electroneuronography; EMG = electromyography; HB = House-Brackmann; VZV = varicella-zoster virus; PRF = pulsed radiofrequency; FFMT = free functional muscle transfer; BTX-A = botulinum toxin type A; NMR = neuromuscular retraining.

The decade ahead will be defined by integration and precision. Multi-omics biomarkers for patient stratification, artificial intelligence models for outcome prediction, patient-specific electrophysiology to guide neuromodulation parameter selection, and advanced neuroimaging for surgical planning will collectively transition PFP care from experience-based empiricism to data-driven precision medicine (47, 166). The ultimate therapeutic goal extends beyond restoration of nerve conduction to encompass quality of life: a symmetrical face and a natural, spontaneous smile. Achieving this goal will require crossing traditional specialty boundaries between neurology, surgery, interventional pain management, and rehabilitation medicine to establish unified, patient-centered care teams (85–87). Figure 2 presents a proposed stage-specific management algorithm that integrates the evidence reviewed herein, providing clinicians with a practical decision framework across the acute, monitoring, recovery, and interventional phases of care.
